# Proteolytic enzymes from *Bromelia antiacantha* as tools for controlled tissue hydrolysis in entomology

**DOI:** 10.1186/2193-1801-2-307

**Published:** 2013-07-09

**Authors:** Laura Macció, Diego Vallés, Ana Maria Cantera

**Affiliations:** Laboratorio de Enzimas Hidrolíticas, Facultad de Ciencias, UdelaR, Montevideo, Uruguay; Catedra de Bioquímica, Facultad de Química, UdelaR, Montevideo, Uruguay; Iguá, 4225, Montevideo, 11400 Uruguay; General Flores, 2124, Montevideo, 11800 Uruguay

**Keywords:** Hydrolysis tissue, Epigyne, Bromelia antiacantha

## Abstract

A crude extract with high proteolytic activity (78.1 EU/mL), prepared from ripe fruit of *Bromelia antiacantha* was used to hydrolyze and remove soft tissues from the epigyne of *Apopyllus iheringi*. This enzymatic extract presented four actives isoforms which have a broad substrate specificity action. Enzyme action on samples was optimized after evaluation under different conditions of pH, enzyme-substrate ratio and time (parameters selected based on previous studies) of treatment (pH 4.0, 6.0 and 8.0 at 42°C with different amount of enzyme). Scanning electron microscopy was used to evaluate conditions resulting in complete digestion of epigyne soft tissues. Optimal conditions for soft tissue removal were 15.6 total enzyme units, pH 6.0 for 18 h at 42°C.

## Introduction

Methods for digesting soft tissues that surround sclerotized organs have been widely used in entomological studies in order to observe and identify small structures by scanning electron microscopy (SEM) or optical microscopy (Muff et al., 2007; Kim et al., 2007; Decae et al., 2007). Detailed morphological studies and identification of subtle differences, needed to resolve taxonomic problems and establish phylogenetic relationships between species (Marusik et al., 2009; Galiano, 2001; Platnick and Jäger, 2008), required the total removal of internal soft tissues for study by SEM (Griswold et al., 2005).

Conventional methods are available for elimination of soft tissues surrounding the entomological organs or structures like epigyne, a highly variable female reproductive structure in spiders (Ramírez 2000; Platnick et al., 1999). The most widely used treatments involve strong alkaline agents such as NaOH, KOH as well as NaClO. These strong chemical agents, however, tend to be aggressive and may damage the surface of the cuticle and a range of other chitinous structures, in essence precluding a comprehensive morphological study (Álvarez-Padilla and Hormiga 2007; Sierwald, 1989).

A recent development for sample pre-treatment to remove soft tissue involves use of proteolytic enzymes. Enzymes are not chemically aggressive and allow for deep cleaning of a sample in which damage to organs under study can be avoided. The only reported use of proteolytic enzymes involved commercially available enzymes normally used for cleaning contact lens, i.e., trypsin and pancreatin (Álvarez-Padilla and Hormiga 2007; Dimitrov et al., 2007). This source of enzymes is expensive, and there are no standardized protocols developed for applications other than intended use. It is also important to consider enzyme stability as well as defining optimal parameters for use such as pH and temperature for hydrolysis of soft animal tissue.

The aim of this study was to evaluate digestion of soft tissue associated with epigyne using proteases present in vegetal extracts under controlled conditions of hydrolysis.

We describe a protocol for enzymatic cleaning of different entomological material in order to be able of being studied by SEM. We selected as enzymatic source proteases present in crude extract of *B. antiacantha* fruit. These enzymes have been studied and characterized for our group previously (Vallés et al., 2007). Based on optimal pH and temperature stability were combined different parameters in order to select the west conditions of soft tissue hydrolysis. In the present work are shown studies of specificity of these enzymes that strengthen the selection made.

## Material & methods

### Chemicals

Bovine serum albumin (BSA), Coomassie Brilliant Blue R-250 (CBB), trichloroacetic acid (TCA), tri (hydroxymethyl) aminomethane (Tris), β-mercaptoethanol, casein, Insulin β chain and Bradford reagent were obtained from Sigma-Aldrich (St. Louis, MO). Acrylamide, Azocasein, Trifluoroacetic acid (TFA) were obtained from Fluka (Milwaukee, WI)

All other chemicals used were of highest purity available from local commercial sources.

### Plants

Ripe fruit from *B. antiacantha* was collected from plants grown in most soil with moderate lighting in the Departmento of Rocha (Uruguay) during early autumn 2011. Fruit was stored at -20°C. Plant fruit was deposited in a botanical collection in the Facultad de Química, UdelaR, Montevideo, Uruguay.

### Animals

The epigyne used were from *A. iheringi*. Specimens were captured in pitfall traps in the suburb of Marindia (Canelones, Uruguay). The animals were subsequently stored in 70% ethanol at room temperature.

### Extraction of proteolytic enzymes

Endocarp of *B. antiacantha* was first separated from the fruit skin and fiber. The endocarp tissue without seeds was then macerated using a mortar, without extracting medium, while maintaining the tissue and fruit juice cold using an ice bath. Endocarp crude extract (CE) was obtained by centrifugation at 6654 *x g* (Sigma 3K18, Osterode am Harz, Germany) for 15 min at 4°C and clarification of the supernatant using Wathman filter paper No. 4. CE was fractionated and stored at -20°C. Protein content in CE was determined by the Bradford method (Bradford, 1976) using BSA as standard.

### Determination of proteolytic activity

Proteolytic activity was determined using azocasein as substrate by a method modified from Andrew and Asenjo (Andrew BA and Asenjo JA, 1986). Briefly, CE was activated for 15 min at 4°C by addition of β-mercaptoethanol to 15 mM. Then, 340 μL of a 1/200 dilution of activated CE, 340 μL of 1% azocasein solution (0.1 M Tris–HCl buffer, pH 7.2) and 340 μL of 0.1M Tris–HCl, pH 7.2, were mixed and incubated 10 min at 37°C. The reaction was stopped by addition of 340 μL 10% TCA, centrifuged for 30 min at 13226 *x g* and absorbance at 337 nm was measured. One enzyme unit (EU) is defined as the amount of enzyme required to produce one unit increase in absorbance at 337 nm under conditions tested.

### Native PAGE and Zymogram

CE was precipitated at -20°C with addition of four volumes acetone and after centrifugation was suspended in 0.1M Tris–HCl, pH 8.8. 10 μL aliquots were loaded onto 8 × 10 × 0.75 cm 10% polyacrylamide minigels. Two gels were run in parallel at 121 V for 1 h. One gel was fixed and stained with CBB R-250, and the other was placed in contact with an agarose gel of the same dimensions that was. Transfer of proteolytic activity to the agarose gel was detected after drying and staining with CBB R-250 (Westergaar et al., 1980).

### Mass spectrometry

A mixture of 1–2 μL crystallization solution (SA or HCCA) and 1 μL of sample were prepared in a 200 μL plastic tube. Volumes of between 0.5-1 μL of this mixture were spotted on MTP 384 target plate polished steel (Bruker Daltonik GmbH) and allowed to evaporate to dryness. Mass spectra were acquired on a Bruker Ultraflex (MALDI-TOF MS) spectrometer equipped with a pulsed nitrogen laser (337 nm), in linear positive ion mode, using a 19 kV acceleration voltage. Molecular mass of protein in CE was determined by MS using SA as matrix.

### Proteolytic enzyme specificity

Hydrolysis reactions of reduced insulin β chain were done in 50 mM Tris–HCl, pH 8.0, 20 mM cysteine and 0.0182: 9.1 nmol enzyme- substrate ratio, at 37°C. The reaction was stopped at 0, 5, 15, 90 min and 16 h by adding 8 μL 0.1% TFA (v/v) to 2 μL of reaction mix. The reaction products at these times were crystallized with HCCA matrix in order to be analyzed by MALDI-TOF MS. Validation and positive control digestions were done using papain as reference enzyme, cysteine protease family C1A. The cleavage sites of the proteolytic enzymes in the insulin β chain were determined using GPMAW software v6.0.

### Epigyne soft tissue digestion

Epigyne structures were removed from animal specimens and stored in 70% ethanol at room temperature prior to preparation. Samples were washed repeatedly with distilled water before treatment with enzymes. The epigyne, (ca. 10 mg each) were incubated in 1.0 mL diluted enzyme solution at pH 4.0, 6.0 or 8.0, and for different times in a 42°C water bath with continuous stirring. The samples were washed and suspended in water, then sonicated (Sonicador ULTRONICS ^UL^16, Teltow, Germany) for 15 min. Subsequently, they were rinsed again and stored in 70% ethanol at room temperature until microscopically analysis. Negative controls included epigyne incubated without added enzyme in distilled water for 22 h at 42°C followed by sonication and storage. The enzymatic removal was evaluated by optical microscopy.

### SEM

Samples were prepared for SEM (JOEL 9500, Tokoyo, Japan) using a standard protocol in which the samples were dehydrated in increasing concentrations of ethanol (50, 75 and 100%), then mounted for examination after coating with gold/palladium alloy.

## Results

CE obtained from fruit of *B. antiacantha* has 78.1 EU/mL proteolytic activity, a protein concentration of 2.53 mg/mL, and thus a specific activity of 30.9 EU/mL proteins.

**Figure 1:**
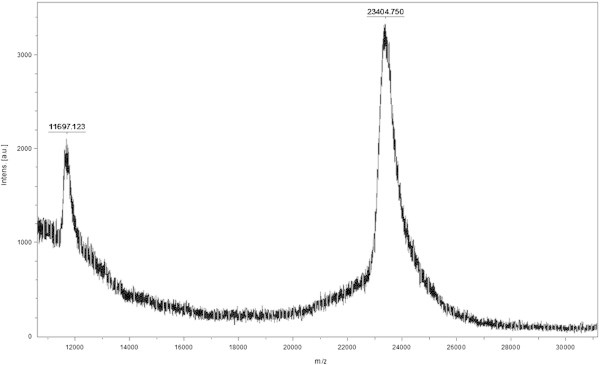
**Mass spectrometry and determination of molecular weight of proteins present in CE of*B. antiacantha*by MALDI-TOF MS/MS.**

**Figure 2:**
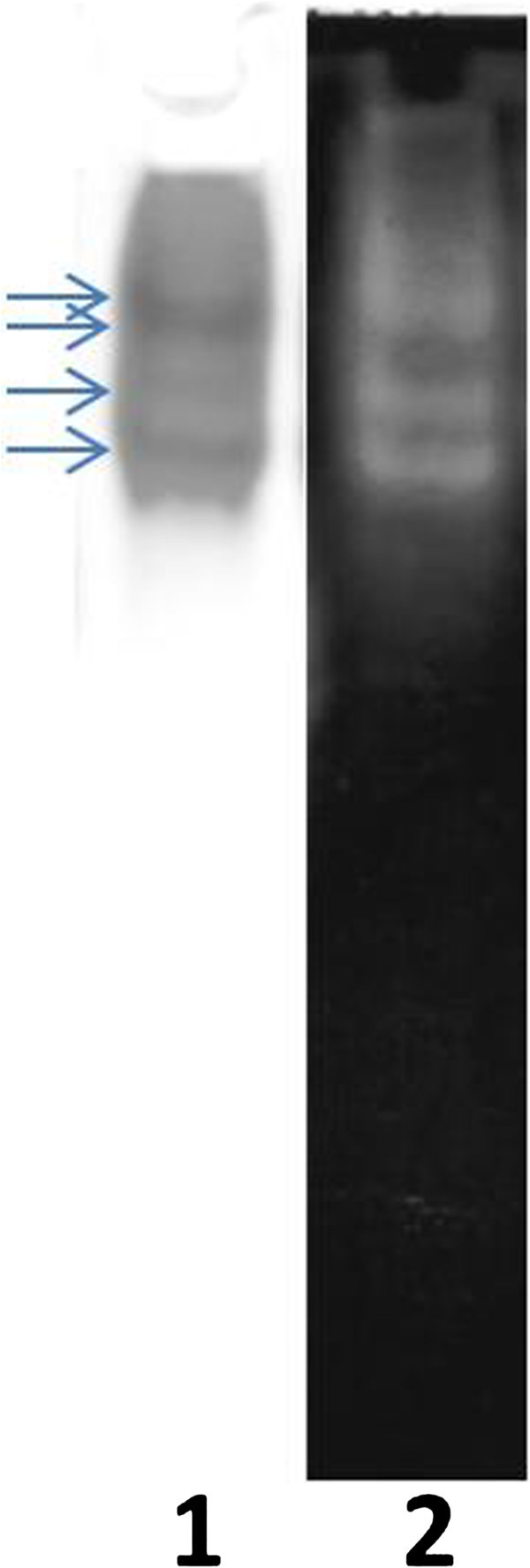
**Native PAGE (lane 1) and Zymogram (lane 2); 25 μg of protein of CE from B.*antiacantha*were loaded.** The arrows represent the proteins bands with proteolytic activity.

Molecular mass determination of the proteins present in the CE by mass spectrometry MALDI-TOF MS/MS shown a single peak of 23404 Da (Figure 1). When the proteins of CE were separated by Native Page, the electrophoresis showed at least 4 bands. All of these proteins bands had proteolytic activity revealed by the zymogram (Figure 2). These results suggest that the four proteases present in CE are isoforms with the same molecular weight.

**Figure 3:**
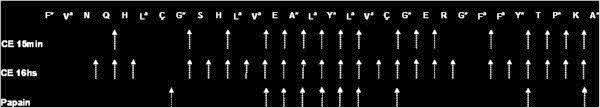
**Determination of cleavage sites of papain and CE enzymes on insulin β chain determined for fragments analysis by MALDI-TOF MS and GPMAW software v6.0.** All insulin β chain cleavage sites are shown with arrows, with positions indicating the P’1 cleavage site. *Hydrofobic residues. ^a^Bulky hydrofobic residues.

Primary specificity of CE proteolytic enzymes was determined by hydrolysis of modified insulin β chain (CBIc). Mass spectrometric analysis of the CBIc digestion fractions at 15 min resulted in the cleavage of more than 10 sites. The analysis of the CBIc digestion fractions for 16 h resulted in more than 20 cleavage sites (Figure 3). Digestion for 16 h with papain (used as positive control of the assay) showed 10 cleavage sites consistent with previous studies (Kaneda et al., 1995).

The proteolytic enzymes of *Bromelia antiacantha* were most frequently found to cleave the CBIc peptid bond adjacent to hydrophobic residues, a characteristic property for this cystein proteases family (Vallés et al., 2007). This broad specificity of CE proteases indicates excellent potential for enzymatic removal of diverse proteinaceous material from different sources.

**Figure 4:**
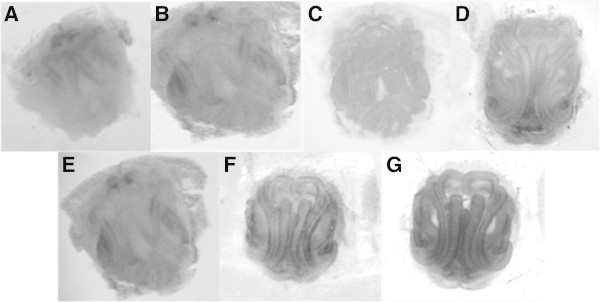
**Photographs taken under increased ventral face of*A. iheringi*epigine subjected to various conditions of pH, CE specific activity and incubation time at 42°C.** Epigine treatment: **A)** incubated in distilled water 22 h, **B)** 2.4 EU_TOT_, pH 4.0, 22 h, **C)** 2.4 EU_TOT_, 0.1 M Tris–HCl, pH 6.0, 22 h, **D)** 2.4 EU_TOT_, 0.1 M Tris–HCl, pH 8.0, 22 h, **E)** 15.6 EU_TOT_, 0.1 M Tris–HCl, pH 8.0, 6 h, **F)** 15.6 EU_TOT_, 0.1 M Tris–HCl, pH 6.0, 6 h and **G)** 15.6 EU_TOT_, 0.1 M Tris–HCl, pH 6.0, 8 h.

Previous studies to determine the optimal temperature and pH for CE proteolytic enzymes showed highest activity at 60°C and between pH 6.0-9.0. The stability range for the activity was highest between 37-55°C and pH 4.0-9.0 (Vallés et al., 2007). Conditions selected for evaluation of optimizing digestion of epigyne soft tissues were pH 4.0, 6.0 and 8.0 at 42°C. Table 1 shows the conditions under which CE was tested in these initial experiments. The epigyne treated under these conditions were evaluated by optical microscopy (Figure 4).

**Table 1:** **Proteolysis conditions (pH, incubation time, and specific activity EU**_**TOT**_**) evaluated for different samples**

Species	pH	Time (hs)	Enzyme unit (total)*
*A. iheringi*	4	22	2,4
	6		
	8		
	6	6	15,6
	8		
	6	8	

In all cases the treatment was done in a water bath at 42°C with continuous stirring. *EU_TOT_ means total activity at the volume of enzyme used in the treatment.

Sonication of epigyne for 15 min without proteolytic enzyme treatment (Epigyne treated only by sonication during 15 min,) did not result in removal of soft tissue (Figure 4a). The large amount of tissue present without added enzyme completely obstructed observation of underlying morphological structure. The soft tissues digestion was evaluated at the same amount of enzymes and different pH. Treatment at pH 4.0 resulted in virtually no removal of soft tissue (Figure 4b). There was also only a partial clearing of soft tissue with enzyme at pH 8.0 (Figure 4d). Adjustment of the pH to 6.0 with the same amount of enzyme resulted in complete digestion of soft tissue including the cleaning of ducts (Figure 4c). The high removal of soft tissues achieved at pH 6.0 is also consistent with previously reported stability and optimum pH reported for these enzymes (Vallés et al., 2007). The large amounts of soft tissue remaining in epigyne enzymatically treated at pH 4.0 and 8.0 obstructed any detail in these structures when examined by optical microscopy made these samples unable to be studied by SEM.

Optimization of enzyme-substrate ratio and the time needed for effective cleaning were further evaluated at pH 6.0 and 8.0. More effective digestion was achieved after 6 h at pH 6.0 compared with the same treatment but at pH 8.0 (Figure 4e and f, respectively). There was, in fact, virtually no difference between enzyme-treated at pH 8.0 and untreated epigyne (Figure 4e y a, respectively). Finally, treatment done at pH 6.0 for 8 h resulted in complete removal of soft tissue from the epigyne structure (Figure 4g). These enzymatic cleaning conditions appeared to completely remove all soft tissue and were thus used for preparing epigyne for study by SEM.

**Figure 5:**
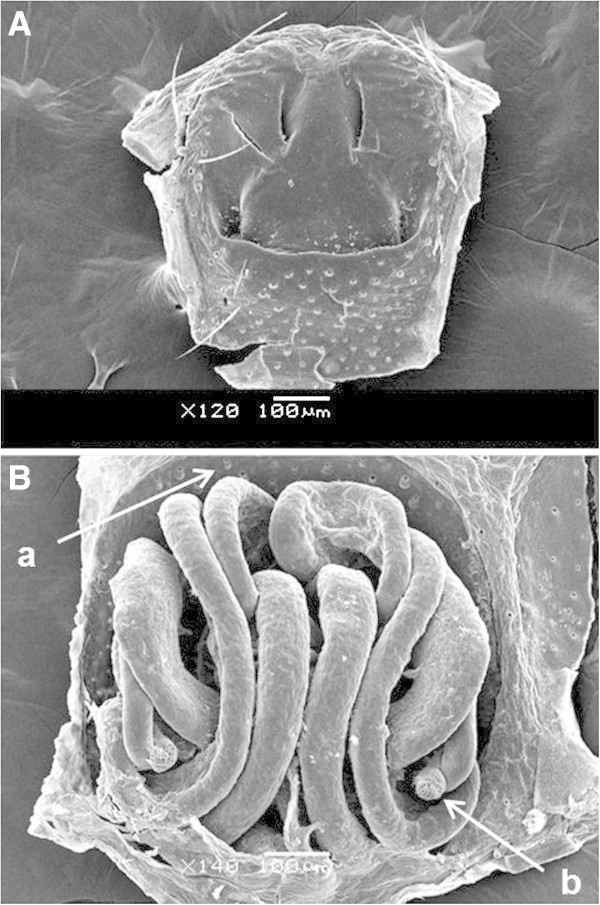
**SEM image of*A. iheringi*epigyne treated for 8 h with 15.6 EU**_**TOT**_**of CE (0.1 M Tris–HCl, pH 6.0 at 42°C).** Image **A** shows dorsal view and image **B** shows ventral view. In figure B can identified two structures: **a)** porous zone and **b)** spermathecae.

**Figure 6:**
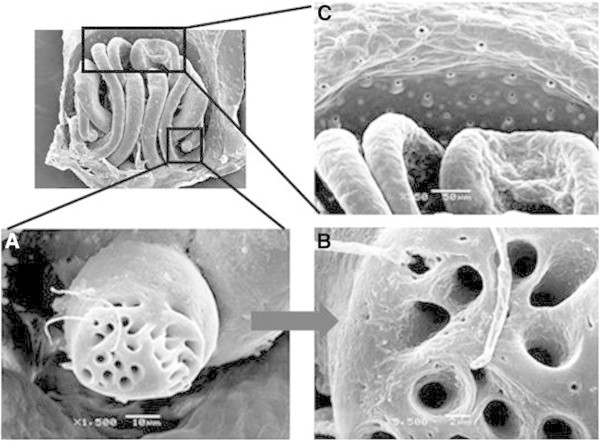
**Spermatheca and porous zone shown at higher magnification: A) right spermathecae B) right spermatheca higher magnification and C) porous zone.**

**Figure 7:**
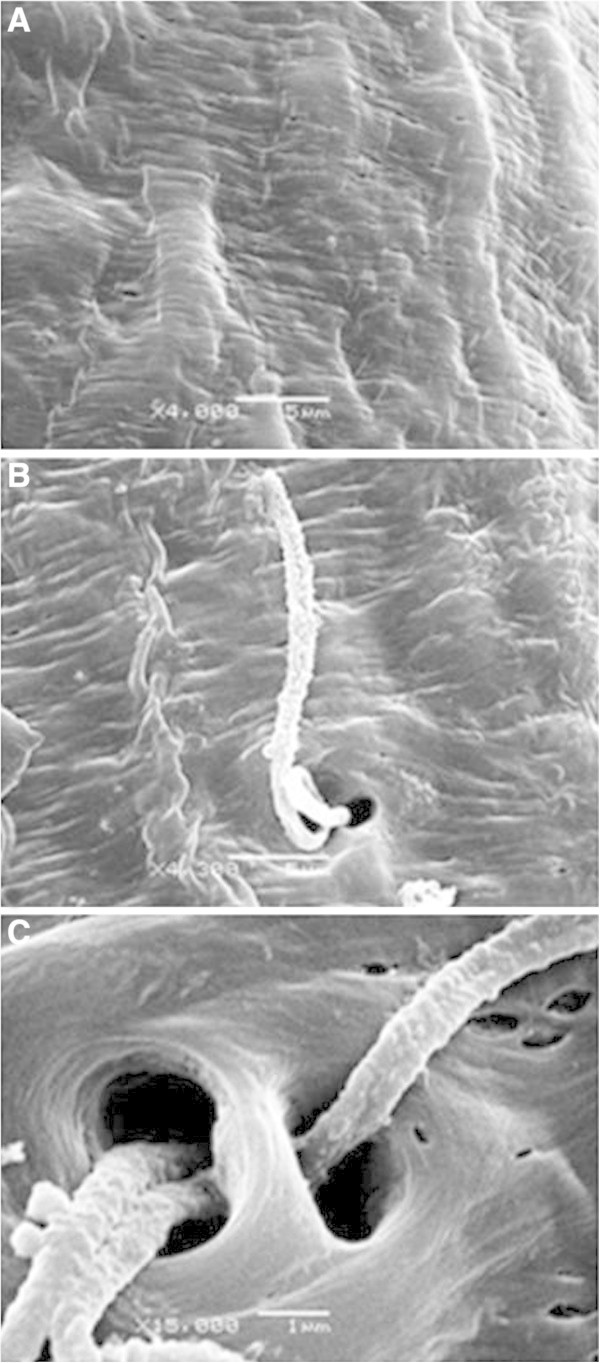
**Surface duct and pore of epigyne shown at higher magnification: A) surface duct B and C) Pore of epigyne duct.**

Despite the great morphological complexity of the epigyne and number of ducts present, the complete removal of soft tissue is of great importance for taxonomic studies of these species (Figure 5). Structural detail of the epigyne in the absence of soft tissue was revealed including porous areas positioned behind the ducts and spermathecae (Figure 6). The spermathecae are internal female structures used for storing sperm after copulation and are very important for resolving systematic differences (Montes de Oca and Pérez-Miles 2009). In addition, some fibrous structures were evident that to our knowledge were not reported previously and are likely of non-protein composition (Figure 7). The cleaning treatment used enabled detailed observation of the chitinous cuticle surface of epigyne without observed damage, and thus providing an alternative to conventional chemical methods (Figures 6 and 7).

The time required for soft tissue removal using this enzymatic approach described here is much shorter than current conventional methods (Álvarez-Padilla and Hormiga 2007; Dimitrov et al., 2007). Furthermore, there was no observable damage to the chitinous cuticle of these structures like it was observed in samples treated with KOH which cuticle surface was digested by the caustic process making the specimen unsuitable for SEM (Álvarez-Padilla and Hormiga 2007). These results suggest the absence of chitinase enzymes in the CE.

The CE of *B. antiacantha* was also effective in removing epigyne soft tissue from two other spiders (*Camillina chilensis* and *Anelosimus studiosus*) using the same conditions as for *A. iheringi* (15.6 UE _TOT_, pH 6.0 for 8 h at 42°C) (Data not shown). There is high variation in both the morphology and complexity of epigyne, and was the case for the three spiders used in this study. These species also likely have significant differences in protein composition of epigyne soft tissues. However, conditions for digestion of these soft tissues (pH, temperature and enzyme activity) were the same. This may be due to the high efficiency in removal of proteinaceous material using the enzymatic extract of *B. antiacantha*. Significant differences in protein content and composition can be expected for other soft tissues in spiders and insects, and thus the method may be generally useful in entomology.

## Conclusions

Crude extract from the ripe fruit of *Bromelia antiacantha* has four proteolytic enzymes isoforms with broad specificity.

These enzymes were very effective in total removal of soft tissue and cleaning of *A. iheringi* epigine for detailed structure analysis. Total elimination of soft tissues from epigyne was competed under optimized conditions (15.6 UE _TOT_, pH 6.0 for 8 h at 42°C).

Was described a novel, fast and economical protocol for cleaning entomological samples for use in SEM.
